# *Commiphora molmol* Modulates Glutamate-Nitric Oxide-cGMP and Nrf2/ARE/HO-1 Pathways and Attenuates Oxidative Stress and Hematological Alterations in Hyperammonemic Rats

**DOI:** 10.1155/2017/7369671

**Published:** 2017-06-28

**Authors:** Ayman M. Mahmoud, Sultan Alqahtani, Sarah I. Othman, Mousa O. Germoush, Omnia E. Hussein, Gadh Al-Basher, Jong Seong Khim, Maha A. Al-Qaraawi, Hanan M. Al-Harbi, Abdulmannan Fadel, Ahmed A. Allam

**Affiliations:** ^1^Physiology Division, Department of Zoology, Faculty of Science, Beni-Suef University, Beni Suef, Egypt; ^2^Department of Endocrinology, Diabetes & Nutrition, Charité-University Medicine Berlin, Germany; ^3^College of Medicine, King Saud Bin Abdulaziz University for Health Science (KSAU-HS), Riyadh, Saudi Arabia; ^4^King Abdullah International Medical Research Center (KAIMRC), Riyadh, Saudi Arabia; ^5^Biology Department, Faculty of Science, Princess Nourah Bint Abdulrahman University, Riyadh, Saudi Arabia; ^6^Biology Department, Faculty of Science, Aljouf University, Sakakah, Aljouf, Saudi Arabia; ^7^Zoology Department, College of Science, King Saud University, Riyadh, Saudi Arabia; ^8^School of Earth and Environmental Sciences & Research Institute of Oceanography, Seoul National University, Seoul, Republic of Korea; ^9^School of Healthcare Science, Manchester Metropolitan University, Manchester, UK; ^10^Zoology Department, Faculty of Science, Beni-Suef University, Beni Suef, Egypt

## Abstract

Hyperammonemia is a serious complication of liver disease and may lead to encephalopathy and death. This study investigated the effects of *Commiphora molmol* resin on oxidative stress, inflammation, and hematological alterations in ammonium chloride- (NH_4_Cl-) induced hyperammonemic rats, with an emphasis on the glutamate-NO-cGMP and Nrf2/ARE/HO-1 signaling pathways. Rats received NH_4_Cl and *C. molmol* for 8 weeks. NH_4_Cl-induced rats showed significant increase in blood ammonia, liver function markers, and tumor necrosis factor-alpha (TNF-*α*). Concurrent supplementation of *C. molmol* significantly decreased circulating ammonia, liver function markers, and TNF-*α* in hyperammonemic rats. *C. molmol* suppressed lipid peroxidation and nitric oxide and enhanced the antioxidant defenses in the liver, kidney, and cerebrum of hyperammonemic rats. *C. molmol* significantly upregulated Nrf2 and HO-1 and decreased glutamine and nitric oxide synthase, soluble guanylate cyclase, and Na^+^/K^+^-ATPase expression in the cerebrum of NH_4_Cl-induced hyperammonemic rats. Hyperammonemia was also associated with hematological and coagulation system alterations. These alterations were reversed by *C. molmol*. Our findings demonstrated that *C. molmol* attenuates ammonia-induced liver injury, oxidative stress, inflammation, and hematological alterations. This study points to the modulatory effect of *C. molmol* on glutamate-NO-cGMP and Nrf2/ARE/HO-1 pathways in hyperammonemia. Therefore, *C. molmol* might be a promising protective agent against hyperammonemia.

## 1. Introduction

Hepatic encephalopathy (HE) is a serious complication of both acute and chronic liver diseases [1, 2]. HE has been estimated to occur in 10–50% of patients with transjugular intrahepatic portosystemic shunt and 30–45% of patients with cirrhosis, whereas minimal HE affects 20–60% of patients with liver disease [3]. Although the pathological mechanism of HE is not fully understood, ammonia is known to play a key role in HE [4]. Ammonia is a known neurotoxin and induces harmful effects to the central nervous system [5]. Blood ammonia level is strongly correlated with the increased risk of HE and is therefore used as a diagnostic marker for encephalopathy [6]. Ammonia is normally detoxified in the liver via urea cycle, and if does not proceed properly, as in cases of liver failure or congenital defect of the urea cycle enzymes, ammonia increases and lead to HE [7].

The brain removes ammonia through glutamine synthesis driven by glutamine synthetase (GS). Therefore, high ammonia levels can increase glutamine synthesis and cause swelling of astrocytes and brain edema [8]. In addition, hyperammonemia can alter the mitochondrial function and neurotransmission and induce oxidative/nitrosative stress [9–11]. Hyperammonemia has been demonstrated to increase the activity of nitric oxide synthase (NOS) and subsequently nitric oxide (NO) production in the brain [12]. High levels of NO were associated with HE, hyperammonemic syndromes, and other disorders without significant neuronal damage [13, 14]. Moreover, ammonia-induced oxidative stress occurs due to increased production of reactive oxygen species (ROS) and subsequent damage of proteins, lipids, and DNA [15]. Previous studies have demonstrated increased ROS production and oxidative stress in hyperammonemia [10, 11, 16]. Hence, counteracting oxidative/nitrosative stress may represent a protective strategy against hyperammonemia-induced brain injury. In this context, several in vitro and in vivo studies have demonstrated the role of nuclear factor erythroid 2-related factor 2 (Nrf2)/antioxidant response element (ARE) signaling pathway as a contributor to the cellular responses to neuronal injury [17–20]. Through binding to the promoter sequence ARE, Nrf2 controls the expression of antioxidant, defensive, and detoxification genes to remove ROS and reactive nitrogen species (RNS) [21]. However, its role in hyperammonemia is still not fully understood.

Excess ammonia reduces glutamate uptake and increases extracellular glutamate levels [22], leading to activation of the N-methyl-D-aspartic acid (NMDA) glutamate receptor in the brain cortex [23]. Consequently, intracellular calcium (Ca^2+^) increases followed by increased NO production and activation of soluble guanylate cyclase (sGC) and subsequently increases cyclic guanosine monophosphate (cGMP) production [24]. Excess ammonia also increases the activity of Na^+^/K^+^-ATPase in the brain [25]; however, the underlying mechanism remains unclear. Increased activity of the brain Na^+^/K^+^-ATPase in hyperammonemia has been demonstrated in multiple previous studies [16, 26].

Current treatments used to reduce ammonia levels are of limited value, and therefore new psychopharmacological agents acting on cellular molecular targets involved in brain neurological alterations are required [27]. Medicinal plants and their derived bioactive phytochemicals have been gaining recognition in the treatment of neurological diseases. *Commiphora molmol* (family *Burseraceae*) is a shrub resembling tropical tree grows in dry forest and produces a resinous exudate called myrrh or oleo-gum resin [28]. Myrrh has been used traditionally for several centuries for the treatment of various diseases and has showed multiple beneficial effects, including antibacterial [29], hypoglycemic [30], anti-inflammatory [31], antioxidant [32], and hepatoprotective efficacies [33]. To the best of our knowledge, nothing has yet been reported on the possible protective effect of *C. molmol* resin against hyperammonemia. Therefore, the present study aimed to investigate the effects of *C. molmol* resin extract against ammonium chloride- (NH_4_Cl-) induced hyperammonemia in rats, pointing to the role of oxidative stress and inflammation, and the glutamate-NO-cGMP and Nrf2/ARE signaling pathways.

## 2. Materials and Methods

### 2.1. Preparation of *C. molmol* Extract and Assay of Radical Scavenging Activity

*C. molmol* resin was purchased from a local herbalist (Harraz Medicinal Plants Co., Cairo, Egypt) and was ground into fine powder. The resin powder was soaked in 90% ethanol for 24 h, filtered, and concentrated using a rotary evaporator at a temperature not exceeding 45°C.

The scavenging activity of *C. molmol* extract against 2,2-diphenyl-1-picrylhydrazyl (DPPH) radicals was assayed according to the method of Kamel et al. [34] using vitamin C as antioxidant reference.

### 2.2. Experimental Animals and Treatments

Eight-week-old male albino Wistar rats (*Rattus norvegicus*) purchased from the Institute of Ophthalmology (Giza, Egypt) were used in the present investigation. The animals were housed in standard cages at 23 ± 2°C with a 12 h dark/light cycle. All animal procedures were approved by the Institutional Animal Ethics Committee of Beni-Suef University (Egypt).

The experimental rats were divided randomly into 4 groups as following:

Group I (control): rats received intraperitoneal (i.p.) injection of 0.9% sodium chloride (NaCl) (3 times/week) and orally administered 0.5% carboxymethyl cellulose (CMC) daily for 8 weeks.

Group II (*C. molmol*): rats received 0.9% NaCl (3 times/week) and orally administered 125 mg/kg body weight *C. molmol* extract [35] suspended in 0.5% CMC daily for 8 weeks.

Group III (NH_4_Cl): rats received 100 mg/kg NH_4_Cl (Sisco Research Laboratories, Mumbai, India) dissolved in 0.9% NaCl (i.p., 3 times/week) [10] and orally administered 0.5% CMC daily for 8 weeks.

Group IV (NH_4_Cl + *C. molmol*): rats received 100 mg/kg NH_4_Cl (i.p., 3 times/week) and orally administered 125 mg/kg body weight *C. molmol* extract suspended in 0.5% CMC daily for 8 weeks.

The doses were adjusted in accordance with changes in the body weight.

### 2.3. Samples Collection and Preparation

By the end of 8 weeks, the animals were fasted overnight and were then sacrificed, and samples were collected for analysis. Whole blood was collected for the assay of ammonia and hematological parameters. Citrated blood samples were used to assay prothrombin time (PT) and activated partial thromboplastin time (aPTT). Other blood samples were left to coagulate for serum preparation.

The liver, kidney, and cerebrum were immediately excised, washed in cold phosphate-buffered saline (PBS), and weighed. Samples from the liver, kidney, and cerebrum were homogenized in cold PBS, centrifuged, and kept frozen for the determination of lipid peroxidation, NO, and antioxidants. Homogenized cerebrum samples were also used to assay glutamine and Na^+^/K^+^-ATPase. Other samples from the cerebrum were collected and kept at −80°C for RNA isolation and Western blot analysis.

### 2.4. Biochemical Assays

#### 2.4.1. Determination of Ammonia, Urea, Liver Function Markers, and TNF-*α*

Blood ammonia was estimated using reagent kit purchased from Spinreact (Spain), according to the method of da Fonseca-Wollheim [36].

Circulating levels of ALT and AST were determined following the method of Schumann and Klauke [37] whereas ALP was assayed according to the method of Wenger et al. [38]. The assay kits were purchased from Spinreact (Spain).

Serum TNF-*α* levels were estimated using specific ELISA kits (R&D Systems, USA), according to the manufacturer's instructions.

#### 2.4.2. Assay of Oxidative Stress and Antioxidant Defenses

Samples from the liver, kidney, and cerebrum were used to assays lipid peroxidation following the method of Preuss et al. [39] and NO using Griess reagent. Reduced glutathione (GSH) [40] and activity of superoxide dismutase (SOD) [41], catalase (CAT) [42], and glutathione peroxidase (GPx) [43] were also determined in the liver, kidney, and cerebrum homogenates.

#### 2.4.3. Assay of Glutamine Level and Na^+^/K^+^-ATPase Activity

Glutamine concentration in the cerebrum of control and experimental rats was determined according to the method of Lund [44]. Na^+^/K^+^-ATPase activity was determined spectrophotometrically through determination of the inorganic phosphate (Pi) liberated from ATP [45]. The concentration of Pi was estimated using reagent kit purchased from Spinreact (Spain), according to the method of Fiske and Subbarow [46].

### 2.5. Hematological Assays

Erythrocytes, hemoglobin content, platelets, total leukocytes, PT, and aPTT were determined using an automated analyzer.

### 2.6. Gene Expression Analysis

Reverse transcriptase polymerase chain reaction (RT-PCR) was used to determine the effect of *C. molmol* extract on the expression of NOS1, sGC, and Na^+^/K^+^-ATPase in the cerebrum of rats. Briefly, total RNA was isolated from the frozen cerebrum samples using TRIzol reagent (Invitrogen, USA). The isolated RNA was quantified and its integrity was checked using formaldehyde-agarose gel electrophoresis. Two *μ*g RNA was used to synthesize cDNA by AMV reverse transcriptase. cDNA was then amplified by Green Master Mix (Fermentas, USA) [47] and the primer set listed in Table 1. The amplified PCR products were loaded into agarose gel and visualized using UV transilluminator. The obtained gel images were scanned and analyzed by ImageJ (version 1.32j, NIH, USA) using *β*-actin as housekeeping gene.

### 2.7. Western Blotting

To test the effect of *C. molmol* on Nrf2 and HO-1 expression in the cerebrum, Western blotting was used as we previously reported [48]. In brief, samples from the cerebrum were homogenized in RIPA buffer with proteinase inhibitors and centrifuged, and protein concentration was determined in the homogenates using Bradford reagent [49]. To determine Nrf2, nuclear proteins were extracted using a commercial kit purchased from Beyotime (China). The samples were electrophoresed on SDS/PAGE, transferred to PVDF membranes, blocked, and incubated with primary antibodies for Nrf2, lamin B, HO-1, and *β*-actin (Santa Cruz Biotechnology, USA). After washing, the membranes were incubated with the secondary antibodies, washed, and then developed using enhanced chemiluminescence kit (Bio-Rad, USA). The intensity of bands was determined and quantified using ImageJ (version 1.32j, NIH, USA).

### 2.8. Statistical Analysis

Results were analyzed by means of one-way ANOVA followed by Tukey's post hoc analysis using GraphPad Prism 5 (La Jolla, CA, USA). The data were presented as means ± standard error of the mean (SEM), and a *P* value <0.05 was considered to be statistically significant.

## 3. Results

### 3.1. Effect of *C. molmol* on Body Weight Changes in Hyperammonemic Rats

**Figure 1:**
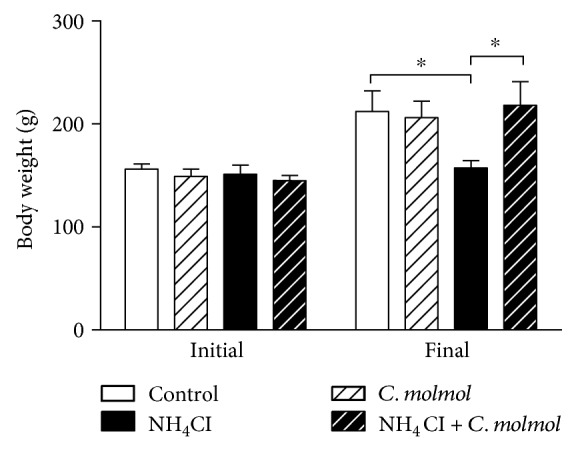
Effect of *C. molmol* resin extract on body weight changes in control and NH_4_Cl-induced hyperammonemic rats. Data are expressed as mean ± SEM (*N* = 6). ^∗^*P* < 0.05.

Initial body weight showed nonsignificant (*P* > 0.05) changes between all experimental groups. Rats received NH_4_Cl administration for 8 weeks which showed a significant (*P* < 0.05) decrease in body weight when compared with the control rats (Figure 1). Concurrent administration of *C. molmol* significantly (*P* < 0.05) improved body weight in NH_4_Cl-induced hyperammonemic rats, while exerting nonsignificant effect when administered to control rats.

### 3.2. Effect of *C. molmol* on Blood Ammonia and Liver Function Markers in Hyperammonemic Rats

Hyperammonemic rats showed a significant (*P* < 0.001) increase in blood ammonia when compared with the control group of rats. Oral administration of *C. molmol* extract to hyperammonemic rats significantly (*P* < 0.001) ameliorated circulating levels of ammonia when compared with the hyperammonemic group (Table 2).

NH_4_Cl-induced hyperammonemia in rats produced a significant (*P* < 0.001) increase in circulating levels of the liver function markers, ALT, AST, and ALP. Treatment of the hyperammonemic rats with *C. molmol* significantly (*P* < 0.001) improved the circulating levels of ALT, AST, and ALP (Table 2). Rats received *C. molmol* alone exhibited nonsignificant changes in blood ammonia, ALT, AST, and ALP when compared with the control rats.

### 3.3. *C. molmol* Decreases Circulating TNF-*α* Levels in Hyperammonemic Rats

Serum levels of the proinflammatory cytokine TNF-*α* showed a significant (*P* < 0.001) increase in NH_4_Cl-induced hyperammonemic rats when compared with the control group (Table 2). Oral supplementation of *C. molmol* resin extract significantly (*P* < 0.01) decreased the circulating levels of TNF-*α* in hyperammonemic rats, with no effect on normal rats.

### 3.4. Effect of *C. molmol* on Lipid Peroxidation, NO, and Antioxidant Defenses in the Liver of Hyperammonemic Rats

**Figure 2:**
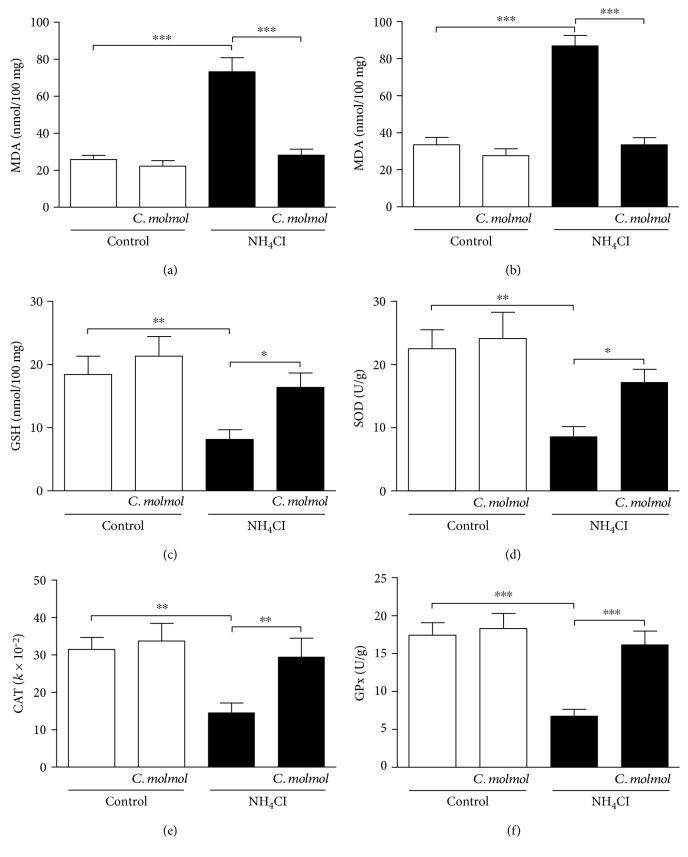
Effect of *C. molmol* resin extract on (a) lipid peroxidation, (b) nitric oxide, (c) GSH, (d) SOD, (e) CAT, and (f) GPx in the liver of NH_4_Cl-induced hyperammonemic rats. Data are expressed as mean ± SEM (*N* = 6). ^∗^*P* < 0.05, ^∗∗^*P* < 0.01, and ^∗∗∗^*P* < 0.001. MDA, malondialdehyde; NO, nitric oxide; GSH, reduced glutathione; SOD, superoxide dismutase; CAT, catalase; GPx, glutathione peroxidase.

Hyperammonemic rats showed a significant (*P* < 0.001) increase in lipid peroxidation (Figure 2(a)) and NO levels (Figure 2(b)) in the liver of rats when compared with the control group. Treatment of the hyperammonemic rats with *C. molmol* markedly decreased liver lipid peroxidation (*P* < 0.001) and NO (*P* < 0.001) levels.

On the other hand, hyperammonemic rats exhibited a significant (*P* < 0.01) decrease in liver GSH content, an effect that was significantly (*P* < 0.05) prevented by *C. molmol* (Figure 2(c)). Similarly, hyperammonemia was associated with significant decline in the activity of SOD (*P* < 0.01; Figure 2(d)), CAT (*P* < 0.01; Figure 2(e)), and GPx (*P* < 0.001; Figure 2(f)) in the liver of rats. Oral administration of *C. molmol* significantly alleviated the activity of SOD (*P* < 0.05), CAT (*P* < 0.01), and GPx (*P* < 0.001) in the liver of hyperammonemic rats.

Oral administration of *C. molmol* to normal rats did not affect liver lipid peroxidation, NO, and antioxidant defenses.

### 3.5. Effect of *C. molmol* on Lipid Peroxidation, NO, and Antioxidant Defenses in the Kidney of Hyperammonemic Rats

**Figure 3:**
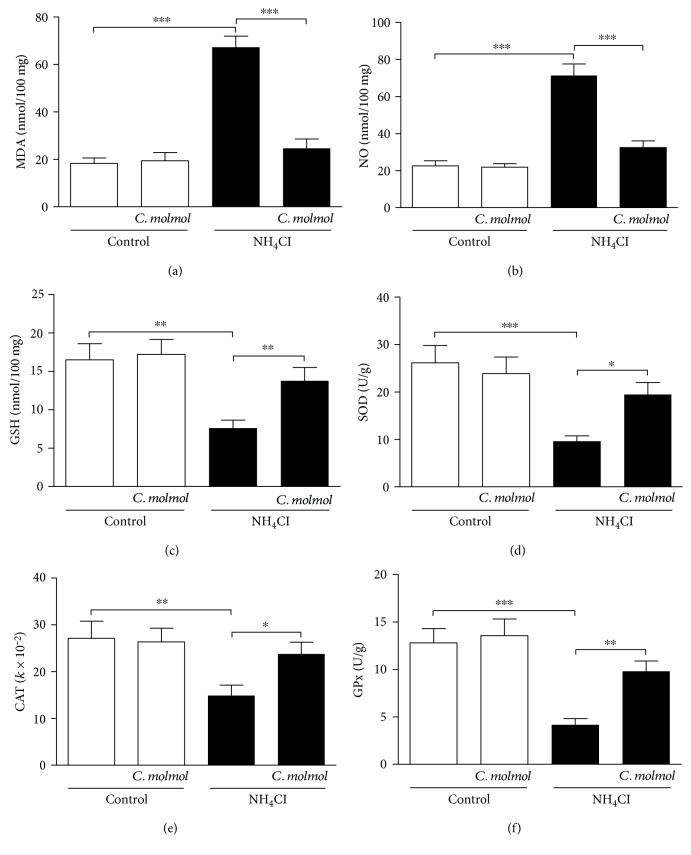
Effect of *C. molmol* resin extract on (a) lipid peroxidation, (b) nitric oxide, (c) GSH, (d) SOD, (e) CAT, and (f) GPx in the kidney of NH_4_Cl-induced hyperammonemic rats. Data are expressed as mean ± SEM (*N* = 6). ^∗^*P* < 0.05, ^∗∗^*P* < 0.01, and ^∗∗∗^*P* < 0.001. MDA, malondialdehyde; NO, nitric oxide; GSH, reduced glutathione; SOD, superoxide dismutase; CAT, catalase; GPx, glutathione peroxidase.

Lipid peroxidation and NO levels showed a significant (*P* < 0.001) increase in the kidney of hyperammonemic rats when compared with the control group. Treatment of the hyperammonemic rats with *C. molmol* significantly (*P* < 0.001) reduced both lipid peroxidation (Figure 3(a)) and NO (Figure 3(b)) in the kidney. *C. molmol* administration to normal rats exerted nonsignificant (*P* > 0.05) effect on kidney lipid peroxidation and NO.

GSH levels showed a significant (*P* < 0.01) decrease in the kidney of hyperammonemic rats when compared with the control rats, an effect that was markedly (*P* < 0.01) prevented by *C. molmol* extract as depicted in Figure 3(c). Similarly, hyperammonemic rats exhibited significantly declined activity of kidney SOD (*P* < 0.001, Figure 3(d)), CAT (*P* < 0.01, Figure 3(e)), and GPx (*P* < 0.001, Figure 3(f)) when compared with the control group. *C. molmol* administration significantly improved the activity of SOD (*P* < 0.05), CAT (*P* < 0.05), and GPx (*P* < 0.01) in kidneys of hyperammonemic rats, with no effect on normal rats.

### 3.6. Effect of *C. molmol* on Lipid Peroxidation, NO, and Antioxidant Defenses in the Cerebrum of Hyperammonemic Rats

**Figure 4:**
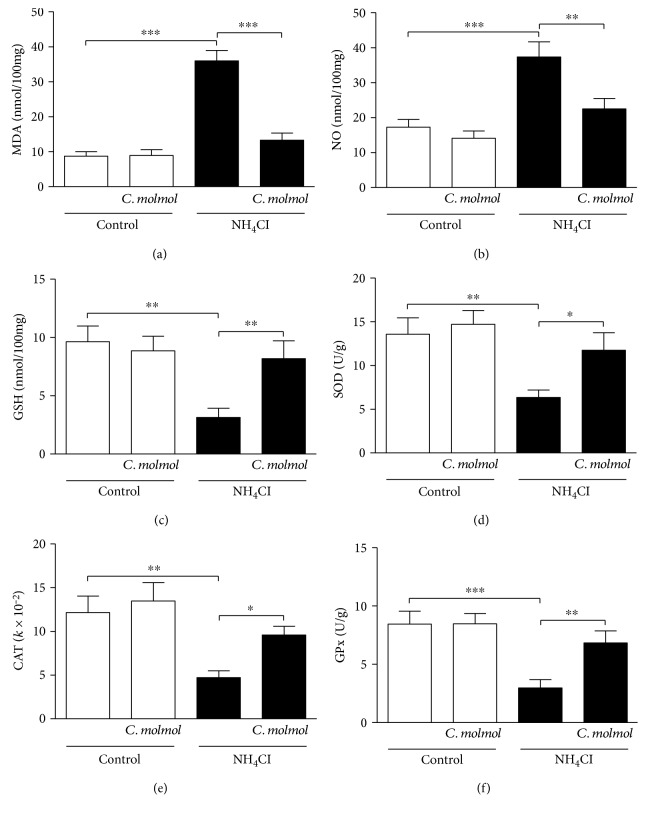
Effect of *C. molmol* resin extract on (a) lipid peroxidation, (b) nitric oxide, (c) GSH, (d) SOD, (e) CAT, and (f) GPx in the cerebrum of NH_4_Cl-induced hyperammonemic rats. Data are expressed as mean ± SEM (*N* = 6). ^∗^*P* < 0.05, ^∗∗^*P* < 0.01, and ^∗∗∗^*P* < 0.001. MDA, malondialdehyde; NO, nitric oxide; GSH, reduced glutathione; SOD, superoxide dismutase; CAT, catalase; GPx, glutathione peroxidase.

NH_4_Cl administration induced a significant (*P* < 0.001) increase in the levels of lipid peroxidation (Figure 4(a)) and NO (Figure 4(b)) in the cerebrum of rats when compared with the control group. In addition, hyperammonemic rats exhibited marked decrease in the cerebral GSH levels (*P* < 0.01; Figure 4(c)), and the activity of SOD (*P* < 0.01; Figure 4(d)), CAT (*P* < 0.01; Figure 4(e)), and GPx (*P* < 0.001; Figure 4(f)). Treatment of the hyperammonemic rats with *C. molmol* significantly decreased lipid peroxidation (*P* < 0.001) and NO (*P* < 0.01) and significantly improved GSH (*P* < 0.01), SOD (*P* < 0.05), CAT (*P* < 0.05), and GPx (*P* < 0.01) in the cerebrum. Oral supplementation of *C. molmol* did not affect lipid peroxidation, NO, and antioxidant defenses in the cerebrum of normal rats.

### 3.7. *C. molmol* Upregulates the Nrf2/ARE/HO-1 Pathway in the Cerebrum of Hyperammonemic Rats

To investigate the effect of *C. molmol* resin extract on the Nrf2/ARE/HO-1 pathway in hyperammonemic rats, the protein expression of Nrf2 and HO-1 was determined in the cerebrum using Western blotting assay.

**Figure 5:**
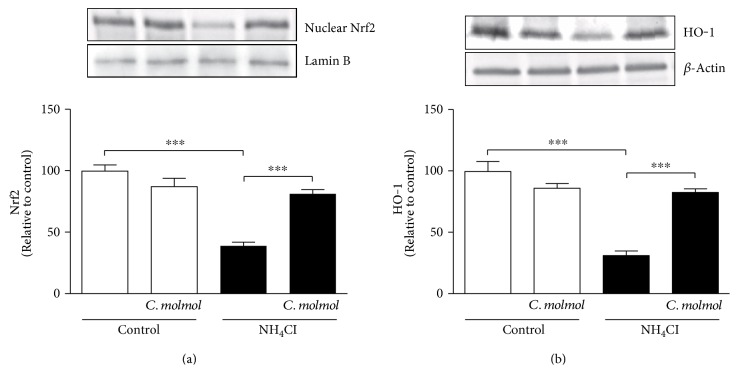
Effect of *C. molmol* resin extract on (a) Nrf2 and (b) HO-1 protein expression in the cerebrum of NH_4_Cl-induced hyperammonemic rats. Data are expressed as mean ± SEM (*N* = 6); ^∗∗∗^*P* < 0.001. Nrf2, nuclear factor erythroid 2-related factor 2; HO-1, hemoxygenase-1.

NH_4_Cl-induced hyperammonemia in rats induced a significant (*P* < 0.001) downregulation of cerebral Nrf2 expression when compared with the control group of rats (Figure 5(a)). Concurrent administration of *C. molmol* resin extract significantly (*P* < 0.001) increased Nrf2 expression in the cerebrum of hyperammonemic rats.

Similarly, HO-1 expression showed a significant (*P* < 0.001) downregulation in the cerebrum of NH_4_Cl-induced hyperammonemic rats when compared with the control rats as depicted in Figure 5(b). *C. molmol* resin supplementation significantly (*P* < 0.001) ameliorated the expression of HO-1 in the cerebrum of hyperammonemic rats. Oral supplementation of *C. molmol* did not induce significant changes in cerebral Nrf2 and HO-1 expression.

### 3.8. *C. molmol* Prevents Hyperammonemia-Associated Hematological Alterations in Rats

To evaluate the effect of *C. molmol* extract on hyperammonemia-associated hematological alteration, RBCs, Hb, WBCs, and platelets were determined in the control and hyperammonemic rats.

**Figure 6:**
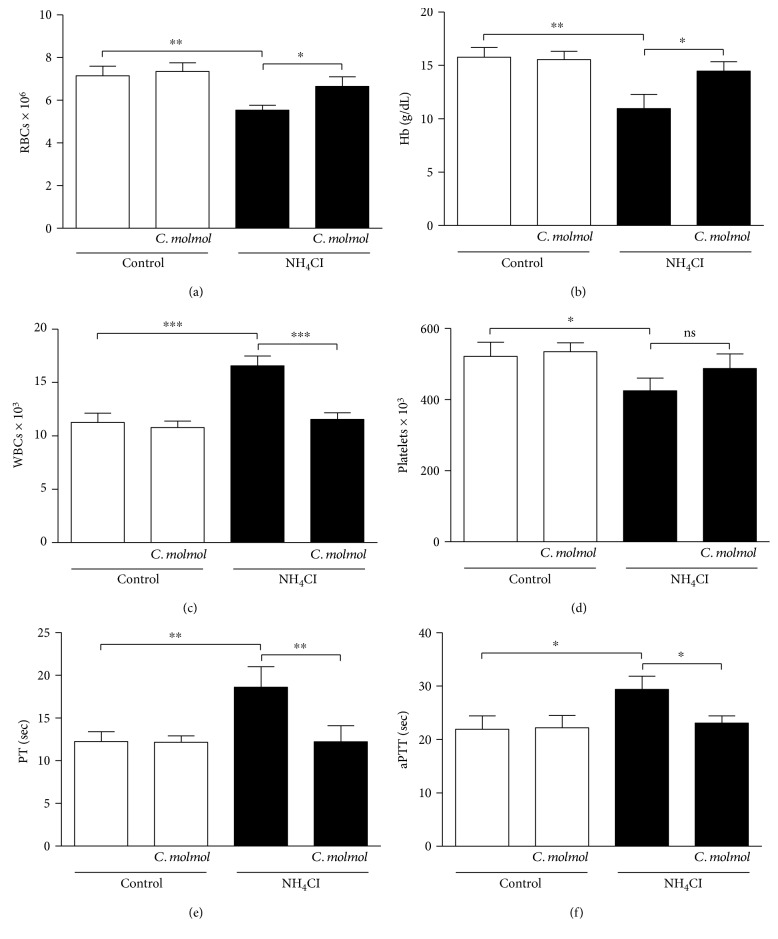
Effect *C. molmol* resin extract on hematologic and coagulation system parameters in NH_4_Cl-induced hyperammonemic rats. Data are expressed as mean ± SEM (*N* = 6). ^∗^*P* < 0.05, ^∗∗^*P* < 0.01, and ^∗∗∗^*P* < 0.001. RBCs, erythrocytes; Hb, hemoglobin; WBCs, leukocytes; PT, prothrombin time; aPTT, activated partial thromboplastin time.

Hyperammonemic rats exhibited a significant (*P* < 0.01) decrease in the number of erythrocytes (Figure 6(a)) and in the Hb content (Figure 6(b)) when compared with the control rats. On the other hand, NH_4_Cl-induced hyperammonemia was associated with significant (*P* < 0.001) leukocytosis (Figure 6(c)). *C. molmol* administration markedly prevented hyperammonemia-induced anemia (*P* < 0.05) and leukocytosis (*P* < 0.001).

Thrombocytopenia was a characteristic feature for hyperammonemia where the NH_4_Cl-induced hyperammonemic rats showed significant (*P* < 0.05) decrease in the number of thrombocytes as compared to the control group (Figure 6(d)). Oral supplementation of *C. molmol* extract to NH_4_Cl-induced hyperammonemic rats did not affect significantly the thrombocytes count.

To examine hyperammonemia-induced changes in the coagulation system and the effect of *C. molmol*, we determined PT and aPTT. Hyperammonemic rats exhibited a significant (*P* < 0.01) prolongation of PT (Figure 6(e)) and aPTT (Figure 6(f)), an effect that was markedly (*P* < 0.01) reversed by *C. molmol*.

Of note, *C. molmol* did not induce any significant changes on hematological parameters of normal rats.

### 3.9. *C. molmol* Downregulates NOS1, sGC, and Na^+^/K^+^-ATPase and Decreases Glutamine in the Cerebrum of Hyperammonemic Rats

**Figure 7:**
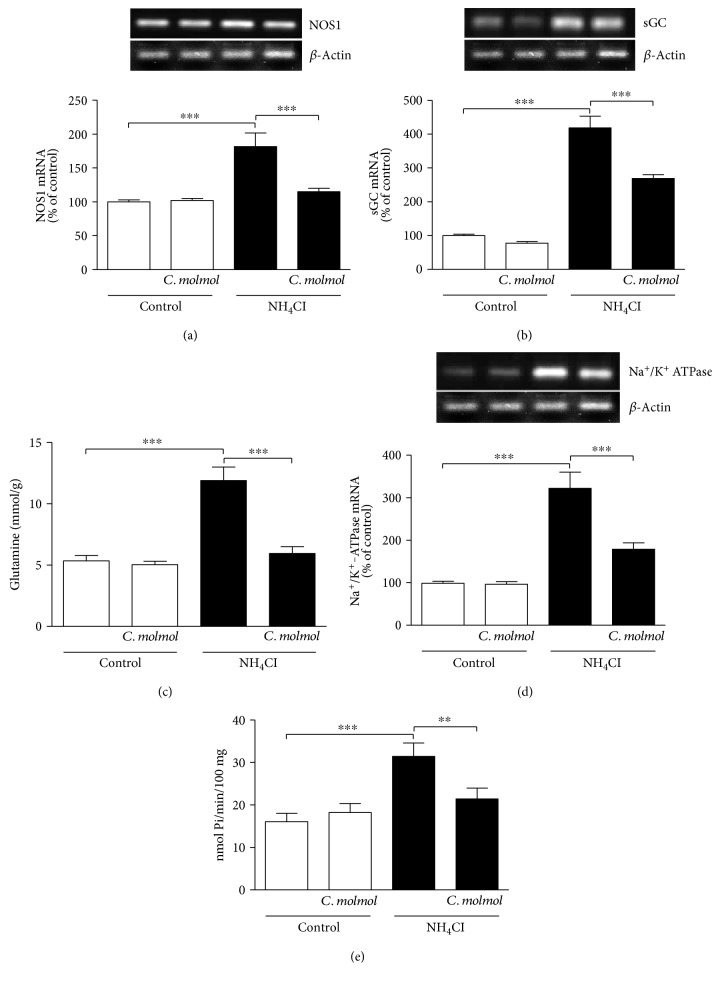
Effect of *C. molmol* resin extract on (a) NOS1 mRNA, (b) sGC mRNA, (c) glutamine levels, (d) Na^+^/K^+^-ATPase mRNA, and (e) Na^+^/K^+^-ATPase activity in the cerebrum of NH_4_Cl-induced hyperammonemic rats. Data are expressed as mean ± SEM (*N* = 6); ^∗∗^*P* < 0.01 and ^∗∗∗^*P* < 0.001. NOS1, neuronal nitric oxide synthase; sGC, soluble guanylate cyclase.

NOS1 mRNA expression showed a significant (*P* < 0.001) increase in the cerebrum of NH_4_Cl-induced hyperammonemic rats when compared with the control group (Figure 7(a)). Concurrent supplementation of *C. molmol* significantly (*P* < 0.001) improved the expression of NOS1 mRNA in the cerebrum of hyperammonemic rats.

Hyperammonemic rats exhibited a significant (*P* < 0.001) upregulation in the expression of sGC mRNA, an effect that was significantly (*P* < 0.001) ameliorated by *C. molmol* (Figure 7(b)). Glutamine levels as well showed a significant (*P* < 0.001) increase in the cerebrum of NH_4_Cl-induced hyperammonemic rats when compared with the control rats (Figure 7(c)). Concurrent administration of *C. molmol* significantly (*P* < 0.001) decreased cerebral glutamine levels in NH_4_Cl-induced hyperammonemic rats.

NH_4_Cl-induced hyperammonemia induced a significant (*P* < 0.001) increase in both the expression and activity Na^+^/K^+^-ATPase in the cerebrum of rats. Oral supplementation of *C. molmol* to hyperammonemic rats significantly improved the expression (*P* < 0.01) and activity (*P* < 0.001) of the cerebral Na^+^/K^+^-ATPase as represented in Figures 7(d) and 7(e), respectively.

Oral supplementation of *C. molmol* did not affect the gene expression of NOS1, sGC, Na^+^/K^+^-ATPase, or glutamine levels in the cerebrum of normal rats.

## 4. Discussion

*C. molmol* has showed multiple therapeutic effects; however, nothing has yet been reported on its protective effect against hyperammonemia. The present study shows for the first time the protective effect of *C. molmol* resin extract against excess ammonia-induced alterations, pointing to the role of Nrf2/HO-1 pathway.

An initial objective of this study was to investigate the protective activity of *C. molmol* resin extract against NH_4_Cl-induced liver injury. The liver plays a central role in detoxification of both endogenous and exogenous toxins. This detoxification capacity is hampered upon liver injury, and the body is exposed to the harmful effects of toxicants. Hyperammonemia occurs as a consequence of liver failure [7]. In the present investigation, increased circulating levels of ammonia indicate liver damage induced by ammonia intoxication in rats as we previously reported [10, 11]. Excess ammonia-induced liver injury was confirmed by increased circulating liver-specific marker enzymes ALT, AST, and ALP. In NH_4_Cl-induced animal model, hyperammonemia occurs as a consequence of liver damage induced by injection of NH_4_Cl. Hyperammonemia induces liver damage which may contribute to or exacerbate hyperammonemia and other alterations resulting from liver damage. We have previously reported increased circulating levels of ALT, AST, and ALP in NH_4_Cl-induced hyperammonemic rats [10]. Elevated liver marker enzymes in serum is an indicator for the assessment of hepatocellular damage [50]. *C. molmol* resin extract significantly ameliorated body weight and decreased blood ammonia levels and circulating levels of ALT, AST, and ALP in hyperammonemic rats. These findings suggest hepatoprotective and membrane-stabilizing potentials of *C. molmol*. Accordingly, *C. molmol* resin protected the liver against ethanol-induced hepatotoxicity in rats and decreased the circulating levels of ALT, AST, and ALP [51]. In addition, treatment with *C. molmol* resin extract decreased circulating ALT and AST in carbon tetrachloride- (CCl_4_-) [52] and D-GalN/LPS-induced [53] liver injury in rats and in a rodent model of chemically induced hepatocarcinogenesis [54]. The declined body weight in NH_4_Cl-induced hyperammonemic rats could be explained by the decreased body fat content. Hyperammonemia has been reported to alter lipid metabolism and significantly decrease body lipid content, leading to declined body weight [55]. Alleviated body weight by *C. molmol* resin extract in this study could be attributed to the ameliorated lipid metabolism. However, further studies are required to better explain the possible role of *C. molmol* in ameliorating body weight in hyperammonemic rats.

Hyperammonemic rats in the present study exhibited a marked increase in the circulating levels of TNF-*α* as we recently reported [11]. Although the precise interaction between inflammation and hyperammonemia is unclear, inflammation appears to play important role in the pathogenesis of HE [56–58]. In this context, studies have showed a strong positive correlation between inflammation and HE [59, 60]. In patients with liver cirrhosis, elevated circulating levels of the proinflammatory cytokine TNF-*α* were recorded [59, 60]. Shawcross et al. [61] proposed the crucial role of inflammation in hyperammonemia-associated neuropsychological alterations. Hence, attenuation of inflammation, particularly mediated by TNF*α*, may reduce or prevent hyperammonemia. This notion is supported by the findings of Chung et al. [62] who showed the ability of indomethacin, a nonsteroidal anti-inflammatory drug (NSAID), to prevent ammonia-induced brain edema after portacaval anastomosis in rats. In addition, we have demonstrated that the ameliorated blood ammonia levels was associated with decreased circulating levels of TNF-*α* in NH_4_Cl-induced hyperammonemia in rats [11].

Here, *C. molmol* resin extract significantly decreased serum levels of TNF-*α*, demonstrating that its anti-inflammatory effect plays a role in attenuating hyperammonemia. The anti-inflammatory efficacy of *C. molmol* has been reported in different studies. In a rat model of formalin-induced hind paw edema, Shalaby and Hammouda [35] showed that *C. molmol* exerted a potent anti-inflammatory effect. This effect has been attributed to the reduced production of prostaglandins as reported by Su et al. [63]. Also, Ahmad et al. [53] showed that myrrh attenuated inflammation in a rat model of D-GalN/LPS-induced hepatic injury. Recently, Fatani et al. [64] showed the anti-inflammatory efficacy of *C. molmol* in acetic acid-induced ulcerative colitis in rats. We have recently demonstrated the potent anti-inflammatory effect of *C. molmol* resin extract in a rat model of chemically induced hepatocarcinogenesis [54]. These studies support the idea that attenuation of inflammation has a role in the protective mechanism of *C. molmol* against hyperammonemia.

Attenuation of oxidative/nitrosative stress is another mechanism we hypothesized to mediate the protective effect of *C. molmol* resin extract against NH_4_Cl-induced hyperammonemia in rats. In animal models, excess ammonia provokes excessive production of ROS [15] which initiate lipid peroxidation. Here, hyperammonemic rats exhibited marked increase in lipid peroxidation levels in the liver, kidney, and cerebrum. Previous research from our lab showed significant increase in lipid peroxidation in the liver, brain, and kidney of NH_4_Cl-induced hyperammonemic rats [10, 11]. Other studies have reported increased lipid peroxidation in animal models of hyperammonemia [16, 65, 66]. Moreover, NH_4_Cl-induced hyperammonemic rats showed a marked increase in NO levels in the liver, kidney, and cerebrum. The increased NO production in the brain is a direct result of the upregulated nNOS in hyperammonemic rats. Accordingly, Ramakrishnan et al. [16] have reported increased expression of nNOS in the brain of NH_4_Cl-induced rats. Hyperammonemia is known to activate nNOS and increase NO production in the brain. The excessive production of NO can induce neuronal damage [12].

Interestingly, *C. molmol* resin significantly alleviated lipid peroxidation in the liver, kidney, and cerebrum of hyperammonemic rats, demonstrating a radical scavenging efficacy. In addition, *C. molmol* significantly decreased NO production which is associated with the decreased nNOS expression. In accordance, supplementation of *C. molmol* to rats with ulcerative colitis [64], ethanol- [51] and lead-induced liver injury [32], and hepatocarcinogenesis [54] significantly decreased lipid peroxidation and NO levels. Moreover, *C. molmol* significantly enhances both enzymatic and nonenzymatic antioxidant defenses in the liver, kidney, and cerebrum of hyperammonemic rats. These antioxidants play key roles in protecting the body against free radicals. Previous studies have reported declined GSH, SOD, CAT, and GPx in the liver, brain, and kidney of NH_4_Cl-induced hyperammonemic rats [10, 11, 16]. Declined antioxidant defense mechanisms in hyperammonemia can aggravate ROS-induced tissue damage. Along with reducing lipid peroxidation and NO, *C. molmol* has been reported to enhance the antioxidant defenses in different tissues of rats [32, 51, 54, 64].

The in vivo antioxidant activity of *C. molmol* resin extract is in positive correlation with the in vitro data. Our findings showed a significant antioxidant and radical scavenging efficacies of *C. molmol* resin extract evidenced by the DPPH assay (Supplementary Figure I available online at https://doi.org/10.1155/2017/7369671). The antioxidant activity of *C. molmol* is due to its rich content of bioactive molecules. Phytochemical analysis of *C. molmol* resin showed the presence of active constituents with antioxidant activity. These bioactive constituents include limonene, m-cresol, eugenol, commiphoric acids, furanosesquiterpenes, pinene, terpenoids, and cuminic aldehyde [67, 68]. In addition, the study of Mahboubi and Kazempour [69] showed the presence of phenolic and flavonoid compounds in *C. molmol* resin ethanolic extract. Phenolic compounds are well known for their potent antioxidant and radical scavenging properties [10, 34, 70, 71].

In addition to its radical scavenging property, we hypothesized that *C. molmol* resin extract can enhance antioxidant defenses and abrogate oxidative stress through activation of Nrf2/HO-1 signaling. The possible involvement of Nrf2 activation in mediating the protective activity of *C. molmol* resin against hyperammonemia has not been previously investigated. The current findings showed a significant decline in Nrf2 expression in the cerebrum of hyperammonemic rats. This downregulation has been markedly reversed following treatment with *C. molmol* resin extract. The results also showed that HO-1 gene expression was upregulated in the cerebrum of hyperammonemic rats treated with *C. molmol* resin extract. These findings highlight that activation of the Nrf2/ARE/HO-1 signaling pathway participates in the neuroprotective effect of *C. molmol* resin extract against hyperammonemia-induced injury. Nrf2 is known to be activated by ROS and then dissociates from Keap1 and translocates to the nucleus where it binds to ARE and activates the transcription of antioxidant and cytoprotective proteins including HO-1, CAT, SOD, and GPx [21]. Although activated by ROS, Nrf2 showed a significant decline in the cerebrum of hyperammonemic rats exhibiting oxidative stress. This was further confirmed by the declined HO-1 expression in the cerebrum of hyperammonemic rats. Moreover, the activity of antioxidant defenses in the cerebrum was strongly correlated with these findings. An explanation for this declined expression of Nrf2 could be the chronic and surplus production of ROS. This notion is supported by our recent findings where we demonstrated downregulation of the Nrf2/ARE/HO-1 pathway in different conditions with excessive production of ROS [34, 48, 72–74]. Furthermore, the anti-inflammatory effect of *C. molmol* resin extract could be attributed, at least in part, to the activation of Nrf2. Multiple studies have demonstrated the anti-inflammatory role of Nrf2. Knockout of Nrf2 has been associated with reduced anti-inflammatory efficacy of the antioxidant curcumin [75]. In addition, activation of Nrf2 blocked the transcription of IL-6 and IL-1*β* in macrophages [76].

The effect of *C. molmol* resin on hematological alterations in hyperammonemia was one of our targets in this study; however, reports about hematological alterations in hyperammonemia are very few. Assessment of hematological parameters represents a powerful tool and an earlier indicator to evaluate the deleterious effects of drugs [77]. In the present study, administration of *C. molmol* to normal rats did not affect the hematological and coagulation system parameters, whereas hyperammonemic rats exhibited anemia, leukocytosis, thrombocytopenia, and prolonged PT and aPTT. Hematological abnormalities occur frequently in liver disease conditions [78]. In support of our findings, Kalaitzakis et al. [79] have demonstrated that HE is related to anemia in liver transplant candidates with cirrhosis. The recorded anemia in hyperammonemic rats could also be a consequence of the increased ROS production. ROS can decrease cellular deformability and damage erythrocytes via induction of membrane lipid peroxidation and rigidity [80]. In addition, liver disease is known to be associated with defects of blood coagulation as a consequence of thrombocytopenia, endothelial dysfunction, and deficiencies of coagulation factors. Low circulating levels of the coagulation factors are associated with prolongation of PT and aPTT [81]. Here, hyperammonemic rats exhibited thrombocytopenia and prolonged PT and aPTT. Furthermore, hyperammonemic rats showed leukocytosis. Accordingly, Choi et al. [82] reported mild leukocytosis in hyperammonemic patients with ornithine carbamoyltransferase deficiency and Aggarwal et al. [78] demonstrated a significant association of leukocytosis with HE. Interestingly, *C. molmol* prevented all hematological alterations in hyperammonemic rats. These findings could be a direct result of the prevention of hyperammonemia and attenuation of liver injury, oxidative stress, and inflammation.

Glutamine is a neutral amino acid and functions normally as ammonia carrier in the CNS [83]. Because the brain does not convert ammonia into urea, ammonia is exclusively removed by GS located in astrocytes. Thus, glutamine synthesis is an essential process for the brain to detoxify excess ammonia in liver failure [84]. In hyperammonemic conditions, the metabolism of ammonia to glutamine is followed by an osmotic disturbance, altered cerebral blood flow, oxidative stress, and edema. Other factors including systemic inflammation may contribute to the excess ammonia-induced cerebral alterations [85]. NH_4_Cl-induced hyperammonemic rats in the present investigation showed a marked increase in cerebral glutamine levels and the expression of sGC. Excess ammonia has been reported to activate NMDA receptors in the brain cortex [23], followed by increased intracellular Ca^2+^, increased NO production, and activation of sGC [24]. Interestingly, *C. molmol* supplemented hyperammonemic rats showed a marked decrease in cerebral glutamine levels and downregulated sGC. These findings could be attributed to the ability of *C. molmol* to decrease ammonia levels, oxidative stress, and nNOS expression. Therefore, the protective effect of *C. molmol* resin extract against hyperammonemia is illustrated, at least in part, through inhibiting NMDA receptors and modulation of the glutamate-NO-cGMP pathway.

Excess ammonia can also alter the ionic shifts and affect the membrane potential of nerve cells. Here, NH_4_Cl-induced hyperammonemic rats exhibited significantly increased expression and activity of the cerebral Na^+^/K^+^-ATPase. Previous studies have showed similar findings in hyperammonemic brain conditions [16, 25, 26]. The exact underlying mechanism of the activated Na^+^/K^+^-ATPase in hyperammonemia is not fully understood. Kosenko et al. [86] proposed that the activation of Na^+^/K^+^-ATPase is a result of decreased phosphorylation by protein kinase C (PKC). In addition, the activity of Na^+^/K^+^-ATPase was increased in cerebral cortex following exposure to millimolar concentrations of NH_4_Cl [87]. *C. molmol* supplementation markedly ameliorated both the expression and the activity of Na^+^/K^+^-ATPase in the cerebrum of hyperammonemic rats. These findings added support to the protective role of *C. molmol* resin against the deleterious effects of hyperammonemia.

In conclusion, our study shows for the first time that *C. molmol* resin extract protects against excess ammonia through attenuation of oxidative stress and inflammation and modulation of the glutamate-NO-cGMP pathway. This investigation also confers information that *C. molmol* may be an effective neuroprotective therapeutic agent with a potential mechanism of upregulation Nrf2/ARE/HO-1 pathway and consequently enhances the antioxidant defenses. *C. molmol* ameliorated both the activity and the expression of Na^+^/K^+^-ATPase and prevented hematological alterations in cases of hyperammonemia and hepatic failure. Therefore, *C. molmol* resin may represent a promising protective agent against hyperammonemia, pending further detailed mechanistic studies. This study may serve as a base for future investigations exploring Nrf2-activating agents as therapeutics for hyperammonemia.

## Supplementary Material

Supplementary Figure I: DPPH radical scavenging activity of C. molmol resin extract. Data are the mean values of triplicate and expressed as mean ± SEM.

## Figures and Tables

**Table 1:** Primers used for RT-PCR.

Gene	GenBank Accession number	Sequence (5′-3′)
NOS1	XM_017598257	F: GGCCCTTTTAATGAGGGTTGC R: TCTGTGCTAAGTAGCCGCTC
sGC	M57405	F: TCACCCCCATACCCTTCTGT R: GGTAGACTCTGTTGCGGCTT
Na^+^/K^+^-ATPase (*Atp1a1*)	NM_012504	F: TGGCATCCGAAGTGCTACAG R: CCAGATCACCAACGACGACA
*β*-Actin (*Actb*)	NM_031144	F: CCGCGAGTACAACCTTCTTG R: CAGTTGGTGACAATGCCGTG

**Table 2:** Effect of *C. molmol* on ammonia, liver function marker enzymes, and TNF-*α* in control and hyperammonemic rats.

	Control	*C. molmol*	NH_4_Cl	NH_4_Cl + *C. molmol*
Ammonia (*μ*mol/dL)	75.30 ± 6.23	72.69 ± 4.89	418.20 ± 18.48^∗∗∗^	136.25 ± 8.79^###^
ALT (U/L)	28.78 ± 4.25	26.50 ± 2.48	64.81 ± 6.23^∗∗∗^	36.12 ± 3.14^###^
AST (U/L)	53.45 ± 4.26	48.16 ± 3.89	132.71 ± 8.49^∗∗∗^	68.51 ± 6.77^###^
ALP (U/L)	82.69 ± 6.13	84.26 ± 5.23	179.18 ± 10.26^∗∗∗^	95.50 ± 7.56^###^
TNF-*α* (pg/mL)	32.60 ± 2.42	35.78 ± 3.88	78.59 ± 5.53^∗∗∗^	48.36 ± 4.33^##^

Data are expressed as mean ± SEM (*N* = 6). ^∗∗∗^*P* < 0.001 versus control, and ##*P* < 0.01 and ###*P* < 0.001 versus NH_4_Cl.

## References

[1] Riordan S. M., Williams R. (1997). Treatment of hepatic encephalopathy. *The New England Journal of Medicine*.

[2] Sturgeon J. P., Shawcross D. L. (2014). Recent insights into the pathogenesis of hepatic encephalopathy and treatments. *Expert Review of Gastroenterology & Hepatology*.

[3] Poordad F. F. (2007). Review article: the burden of hepatic encephalopathy. *Alimentary Pharmacology & Therapeutics*.

[4] Romero-Gomez M., Montagnese S., Jalan R. (2015). Hepatic encephalopathy in patients with acute decompensation of cirrhosis and acute-on-chronic liver failure. *Journal of Hepatology*.

[5] Albrecht J., Norenberg M. D. (2006). Glutamine: a Trojan horse in ammonia neurotoxicity. *Hepatology*.

[6] Bernal W., Hall C., Karvellas C. J., Auzinger G., Sizer E., Wendon J. (2007). Arterial ammonia and clinical risk factors for encephalopathy and intracranial hypertension in acute liver failure. *Hepatology*.

[7] Machado M. C., Pinheiro da Silva F. (2014). Hyperammonemia due to urea cycle disorders: a potentially fatal condition in the intensive care setting. *Journal of Intensive Care*.

[8] Chung M. K., Martin D. O., Sprecher D. (2001). C-reactive protein elevation in patients with atrial arrhythmias: inflammatory mechanisms and persistence of atrial fibrillation. *Circulation*.

[9] Norenberg M. D., Jayakumar A. R., Rama Rao K. V. (2004). Oxidative stress in the pathogenesis of hepatic encephalopathy. *Metabolic Brain Disease*.

[10] Mahmoud A. M. (2012). Influence of rutin on biochemical alterations in hyperammonemia in rats. *Experimental and Toxicologic Pathology*.

[11] Mahmoud A. M., Germoush M. O., Soliman A. S. (2014). *Ruta graveolens* mitigates ammonium chloride-induced hyperammonemia by modulating antioxidant status and pro-inflammatory cytokines. *Life Science Journal*.

[12] Kosenko E., Kaminsky Y., Stavroskaya I. G., Felipo V. (2000). Alteration of mitochondrial calcium homeostasis by ammonia-induced activation of NMDA receptors in rat brain in vivo. *Brain Research*.

[13] Minana M. D., Corbalan R., Montoliu C., Teng C. M., Felipo V. (1999). Chronic hyperammonemia in rats impairs activation of soluble guanylate cyclase in neurons and in lymphocytes: a putative peripheral marker for neurological alterations. *Biochemical and Biophysical Research Communications*.

[14] Munoz M. D., Monfort P., Gaztelu J. M., Felipo V. (2000). Hyperammonemia impairs NMDA receptor-dependent long-term potentiation in the CA1 of rat hippocampus in vitro. *Neurochemical Research*.

[15] Lopez-Alarcon C., Denicola A. (2013). Evaluating the antioxidant capacity of natural products: a review on chemical and cellular-based assays. *Analytica Chimica Acta*.

[16] Ramakrishnan A., Vijayakumar N., Renuka M. (2016). Naringin regulates glutamate-nitric oxide cGMP pathway in ammonium chloride induced neurotoxicity. *Biomedicine & Pharmacotherapy*.

[17] Calkins M. J., Jakel R. J., Johnson D. A., Chan K., Kan Y. W., Johnson J. A. (2005). Protection from mitochondrial complex II inhibition in vitro and in vivo by Nrf2-mediated transcription. *Proceedings of the National Academy of Sciences of the United States of America*.

[18] Shih A. Y., Li P., Murphy T. H. (2005). A small-molecule-inducible Nrf2-mediated antioxidant response provides effective prophylaxis against cerebral ischemia in vivo. *The Journal of Neuroscience*.

[19] Satoh T., Kosaka K., Itoh K. (2008). Carnosic acid, a catechol-type electrophilic compound, protects neurons both in vitro and in vivo through activation of the Keap1/Nrf2 pathway via S-alkylation of targeted cysteines on Keap1. *Journal of Neurochemistry*.

[20] Yang C., Zhang X., Fan H., Liu Y. (2009). Curcumin upregulates transcription factor Nrf2, HO-1 expression and protects rat brains against focal ischemia. *Brain Research*.

[21] Itoh K., Chiba T., Takahashi S. (1997). An Nrf2/small Maf heterodimer mediates the induction of phase II detoxifying enzyme genes through antioxidant response elements. *Biochemical and Biophysical Research Communications*.

[22] Monfort P., Munoz M. D., ElAyadi A., Kosenko E., Felipo V. (2002). Effects of hyperammonemia and liver failure on glutamatergic neurotransmission. *Metabolic Brain Disease*.

[23] Hermenegildo C., Monfort P., Felipo V. (2000). Activation of N-methyl-D-aspartate receptors in rat brain in vivo following acute ammonia intoxication: characterization by in vivo brain microdialysis. *Hepatology*.

[24] Montoliu C., Llansola M., Monfort P. (2001). Role of nitric oxide and cyclic GMP in glutamate-induced neuronal death. *Neurotoxicity Research*.

[25] Ribeiro C. A., Hickmann F. H., Wajner M. (2011). Neurochemical evidence that 3-methylglutaric acid inhibits synaptic Na^+^,K^+^−ATPase activity probably through oxidative damage in brain cortex of young rats. *International Journal of Developmental Neuroscience*.

[26] Renuka M., Vijayakumar N., Ramakrishnan A. (2016). Chrysin, a flavonoid attenuates histological changes of hyperammonemic rats: a dose dependent study. *Biomedicine & Pharmacotherapy*.

[27] Gonzalez-Usano A., Cauli O., Agusti A., Felipo V. (2014). Pregnenolone sulfate restores the glutamate-nitric-oxide-cGMP pathway and extracellular GABA in cerebellum and learning and motor coordination in hyperammonemic rats. *ACS Chemical Neuroscience*.

[28] Hanus L. O., Rezanka T., Dembitsky V. M., Moussaieff A. (2005). Myrrh—Commiphora chemistry. *Biomedical Papers of the Medical Faculty of the University Palacky, Olomouc, Czech Republic*.

[29] Rahman M. M., Garvey M., Piddock L. J., Gibbons S. (2008). Antibacterial terpenes from the oleo-resin of Commiphora molmol (Engl.). *Phytotherapy Research*.

[30] Salama A., Ibrahim W., El-Nimr T., Abd El M. A., Tousson E. (2014). Effect of myrrh extract (Mirazid®) on experimentally diabetic rats. *Pharmacologia Pharmacologia*.

[31] Tipton D. A., Lyle B., Babich H., Dabbous M. (2003). In vitro cytotoxic and anti-inflammatory effects of myrrh oil on human gingival fibroblasts and epithelial cells. *Toxicology In Vitro*.

[32] Ashry K. M., El-Sayed Y. S., Khamiss R. M., El-Ashmawy I. M. (2010). Oxidative stress and immunotoxic effects of lead and their amelioration with myrrh (Commiphora molmol) emulsion. *Food and Chemical Toxicology*.

[33] El-Shahat M., El-Abd S., Alkafafy M., El-Khatib G. (2012). Potential chemoprevention of diethylnitrosamine-induced hepatocarcinogenesis in rats: myrrh (Commiphora molmol) vs. turmeric (*Curcuma longa*). *Acta Histochemica*.

[34] Kamel E. M., Mahmoud A. M., Ahmed S. A., Lamsabhi A. M. (2016). A phytochemical and computational study on flavonoids isolated from *Trifolium resupinatum* L. and their novel hepatoprotective activity. *Food & Function*.

[35] Shalaby M. A., Hammouda A. A. (2014). Analgesic, anti-inflammatory and anti-hyperlipidemic activities of Commiphora molmol extract (myrrh). *Journal of Intercultural Ethnopharmacology*.

[36] da Fonseca-Wollheim F. (1990). Preanalytical increase of ammonia in blood specimens from healthy subjects. *Clinical Chemistry*.

[37] Schumann G., Klauke R. (2003). New IFCC reference procedures for the determination of catalytic activity concentrations of five enzymes in serum: preliminary upper reference limits obtained in hospitalized subjects. *Clinica Chimica Acta*.

[38] Wenger C., Kaplan A., Rubaltelli F. F., Hammerman C. (1984). Alkaline phosphatase. *Clinical Chemistry*.

[39] Preuss H. G., Jarrell S. T., Scheckenbach R., Lieberman S., Anderson R. A. (1998). Comparative effects of chromium, vanadium and *Gymnema sylvestre* on sugar-induced blood pressure elevations in SHR. *Journal of the American College of Nutrition*.

[40] Beutler E., Duron O., Kelly B. M. (1963). Improved method for the determination of blood glutathione. *The Journal of Laboratory and Clinical Medicine*.

[41] Marklund S., Marklund G. (1974). Involvement of the superoxide anion radical in the autoxidation of Pyrogallol and a convenient assay for superoxide dismutase. *FEBS European Journal of Biochemistry*.

[42] Cohen G., Dembiec D., Marcus J. (1970). Measurement of catalase activity in tissue extracts. *Analytical Biochemistry Analytical Biochemistry*.

[43] Matkovics B., Szabo L., Varga I. S. (1998). Determination of enzyme activities in lipid peroxidation and glutathione pathways (in Hungarian). *Laboratoriumi Diagnosztika*.

[44] Lund P., Bergmeyer H. U. (1986). L-glutamine and L-glutamate: UV method with glutaminase and glutamate dehydrogenase. *Methods in Enzymatic Analysis*.

[45] Rauchova H., Drahota Z., Koudelova J. (1999). The role of membrane fluidity changes and thiobarbituric acid-reactive substances production in the inhibition of cerebral cortex Na^+^/K^+^−ATPase activity. *Physiological Research*.

[46] Fiske C., Subbarow Y. (1925). The colourimetric determination of phosphorus. *Journal of Biological Chemistry*.

[47] Mahmoud A. M. (2013). Hematological alterations in diabetic rats - role of adipocytokines and effect of citrus flavonoids. *EXCLI Journal*.

[48] Mahmoud A. M., Germoush M. O., Alotaibi M. F., Hussein O. E. (2017). Possible involvement of Nrf2 and PPARgamma up-regulation in the protective effect of umbelliferone against cyclophosphamide-induced hepatotoxicity. *Biomedicine & Pharmacotherapy*.

[49] Bradford M. M. (1976). A rapid and sensitive method for the quantitation of microgram quantities of protein utilizing the principle of protein-dye binding. *Analytical Biochemistry*.

[50] Ramaiah S. K. (2007). A toxicologist guide to the diagnostic interpretation of hepatic biochemical parameters. *Food and Chemical Toxicology*.

[51] Al-Rejaie S. S. (2012). Effect of oleo-gum-resin on ethanol-induced hepatotoxicity in rats. *Journal of Medical Sciences (Faisalabad)*.

[52] Alm-Eldeen A. A., El-Naggar S. A., El-Boray K. F., Elgebaly H. A., Osman I. H. (2016). Protective role of commiphora molmol extract against liver and kidney toxicity induced by carbon tetrachloride in mice. *Tropical Journal of Pharmaceutical Research*.

[53] Ahmad A., Raish M., Ganaie M. A. (2015). Hepatoprotective effect of Commiphora myrrha against d-GalN/LPS-induced hepatic injury in a rat model through attenuation of pro inflammatory cytokines and related genes. *Pharmaceutical Biology*.

[54] Mahmoud A. M., Zaki A. R., Hassan M. E., Mostafa-Hedeab G. (2017). Commiphora molmol resin attenuates diethylnitrosamine/phenobarbital-induced hepatocarcinogenesis by modulating oxidative stress, inflammation, angiogenesis and Nrf2/ARE/HO-1 signaling. *Chemico-Biological Interactions*.

[55] Minana M. D., Felipo V., Wallace R., Grisolia S. (1989). Hyperammonemia decreases body fat content in rat. *FEBS Letters*.

[56] Shawcross D., Jalan R. (2005). The pathophysiologic basis of hepatic encephalopathy: central role for ammonia and inflammation. *Cellular and Molecular Life Sciences*.

[57] Shawcross D. L., Wright G., Olde Damink S. W., Jalan R. (2007). Role of ammonia and inflammation in minimal hepatic encephalopathy. *Metabolic Brain Disease*.

[58] Shawcross D. L., Wright G. A., Stadlbauer V. (2008). Ammonia impairs neutrophil phagocytic function in liver disease. *Hepatology*.

[59] Odeh M., Sabo E., Srugo I., Oliven A. (2004). Serum levels of tumor necrosis factor-alpha correlate with severity of hepatic encephalopathy due to chronic liver failure. *Liver International*.

[60] Odeh M., Sabo E., Srugo I., Oliven A. (2005). Relationship between tumor necrosis factor-alpha and ammonia in patients with hepatic encephalopathy due to chronic liver failure. *Annals of Medicine*.

[61] Shawcross D. L., Davies N. A., Williams R., Jalan R. (2004). Systemic inflammatory response exacerbates the neuropsychological effects of induced hyperammonemia in cirrhosis. *Journal of Hepatology*.

[62] Chung C., Gottstein J., Blei A. T. (2001). Indomethacin prevents the development of experimental ammonia-induced brain edema in rats after portacaval anastomosis. *Hepatology*.

[63] Su S., Hua Y., Wang Y. (2012). Evaluation of the anti-inflammatory and analgesic properties of individual and combined extracts from Commiphora myrrha, and Boswellia carterii. *Journal of Ethnopharmacology*.

[64] Fatani A. J., Alrojayee F. S., Parmar M. Y., Abuohashish H. M., Ahmed M. M., Al-Rejaie S. S. (2016). Myrrh attenuates oxidative and inflammatory processes in acetic acid-induced ulcerative colitis. *Experimental and Therapeutic Medicine*.

[65] Dakshayani K. B., Velvizhi S., Subramanian P. (2002). Effects of ornithine alpha-ketoglutarate on circulatory antioxidants and lipid peroxidation products in ammonium acetate treated rats. *Annals of Nutrition & Metabolism*.

[66] Kharoubi O., Slimani M., Aoues A., Seddik L. (2008). Prophylactic effects of wormwood on lipid peroxidation in an animal model of lead intoxication. *Indian Journal of Nephrology*.

[67] Chevallier A. (1996). *The Encyclopedia of Medicinal Plants*.

[68] Su S. L., Duan J. A., Tang Y. P. (2009). Isolation and biological activities of neomyrrhaol and other terpenes from the resin of Commiphora myrrha. *Planta Medica*.

[69] Mahboubi M., Kazempour N. (2016). The antimicrobial and antioxidant activities of Commiphora molmol extracts. *Biharean Biologist*.

[70] Mahmoud A. M. (2014). Hesperidin protects against cyclophosphamide-induced hepatotoxicity by upregulation of PPARgamma and abrogation of oxidative stress and inflammation. *Canadian Journal of Physiology and Pharmacology*.

[71] Mahmoud A. M., Ashour M. B., Abdel-Moneim A., Ahmed O. M. (2012). Hesperidin and naringin attenuate hyperglycemia-mediated oxidative stress and proinflammatory cytokine production in high fat fed/streptozotocin-induced type 2 diabetic rats. *Journal of Diabetes and its Complications*.

[72] Mahmoud A. M., Al Dera H. S. (2015). 18*β*-Glycyrrhetinic acid exerts protective effects against cyclophosphamide-induced hepatotoxicity: potential role of PPAR*γ* and Nrf2 upregulation. *Genes & Nutrition*.

[73] Abd El-Twab S. M., Hozayen W. G., Hussein O. E., Mahmoud A. M. (2016). 18beta-Glycyrrhetinic acid protects against methotrexate-induced kidney injury by up-regulating the Nrf2/ARE/HO-1 pathway and endogenous antioxidants. *Renal Failure*.

[74] Mahmoud A. M., Wilkinson F. L., Jones A. M. (2017). A novel role for small molecule glycomimetics in the protection against lipid-induced endothelial dysfunction: involvement of Akt/eNOS and Nrf2/ARE signaling. *Biochimica et Biophysica Acta*.

[75] Boyanapalli S. S., Paredes-Gonzalez X., Fuentes F. (2014). Nrf2 knockout attenuates the anti-inflammatory effects of phenethyl isothiocyanate and curcumin. *Chemical Research in Toxicology*.

[76] Kobayashi E. H., Suzuki T., Funayama R. (2016). Nrf2 suppresses macrophage inflammatory response by blocking proinflammatory cytokine transcription. *Nature Communications*.

[77] Alya A., Ines D. B., Montassar L., Najoua G., Saloua E. F. (2015). Oxidative stress, biochemical alterations, and hyperlipidemia in female rats induced by lead chronic toxicity during puberty and post puberty periods. *Iranian Journal of Basic Medical Sciences*.

[78] Aggarwal H., Rao U. A., Singla S., Dabas G. (2015). A study on hematological spectrum in patients with alcoholic liver cirrhosis. *Journal of Clinical and Experimental Hepatology*.

[79] Kalaitzakis E., Josefsson A., Castedal M. (2013). Hepatic encephalopathy is related to anemia and fat-free mass depletion in liver transplant candidates with cirrhosis. *Scandinavian Journal of Gastroenterology*.

[80] Kolanjiappan K., Manoharan S., Kayalvizhi M. (2002). Measurement of erythrocyte lipids, lipid peroxidation, antioxidants and osmotic fragility in cervical cancer patients. *Clinica Chimica Acta*.

[81] Caldwell S. H., Hoffman M., Lisman T. (2006). Coagulation disorders and hemostasis in liver disease: pathophysiology and critical assessment of current management. *Hepatology*.

[82] Choi D. E., Lee K. W., Shin Y. T., Na K. R. (2012). Hyperammonemia in a patient with late-onset ornithine carbamoyltransferase deficiency. *Journal of Korean Medical Science*.

[83] Albrecht J., Dolinska M. (2001). Glutamine as a pathogenic factor in hepatic encephalopathy. *Journal of Neuroscience Research*.

[84] Dejong C. H., Deutz N. E., Soeters P. B. (1996). Ammonia and glutamine metabolism during liver insufficiency: the role of kidney and brain in interorgan nitrogen exchange. *Scandinavian Journal of Gastroenterology. Supplement*.

[85] Vaquero J., Butterworth R. F. (2007). Mechanisms of brain edema in acute liver failure and impact of novel therapeutic interventions. *Neurological Research*.

[86] Kosenko E., Kaminsky Y., Grau E. (1994). Brain ATP depletion induced by acute ammonia intoxication in rats is mediated by activation of the NMDA receptor and Na^+^,K^+^-ATPase. *Journal of Neurochemistry*.

[87] Sadasivudu B., Rao T. I., Murthy C. R. (1977). Acute metabolic effects of ammonia in mouse brain. *Neurochemical Research*.

