# Supplementary material for: Commiphora molmol Modulates Glutamate-Nitric Oxide-cGMP and Nrf2/ARE/HO-1 Pathways and Attenuates Oxidative Stress and Hematological Alterations in Hyperammonemic Rats
Source: Oxid Med Cell Longev. 2017 Jun 28;2017:7369671. doi: 10.1155/2017/7369671 (PMC5506469; doi:10.1155/2017/7369671)
Supplement: Supplementary file 1 — Supplementary Figure I: DPPH radical scavenging activity of C. molmol resin extract. Data are the mean values of triplicate and expressed as mean ± SEM. [file 7369671.f1.docx]

**Supplementary Material**

***Commiphora molmol* modulates glutamate-nitric oxide-cGMP and Nrf2/ARE/HO-1 pathways and attenuates oxidative stress and hematological alterations in hyperammonemic rats**

**Ayman M. Mahmoud^1,2*^, Sultan Alqahtani^3,4^, Sarah I. Othman^5^, Mousa O. Germoush^6^, Omnia E. Hussein^1^, Gadh Al-Basher^7^, Jong Seong Khim^8^, Maha A. Al-Qaraawi^5^, Hanan M. Al-Harbi^5^, Abdulmannan Fadel^9^, Ahmed A. Allam^7, 10^**

^1^Physiology Division, Department of Zoology, Faculty of Science, Beni-Suef University, Beni-Suef, Egypt.

^2^Department of Endocrinology, Diabetes & Nutrition, Charité-University Medicine Berlin, Berlin, Germany.

^3^College of Medicine, King Saud bin Abdulaziz University for Health Science (KSAU-HS), Riyadh, Saudi Arabia.

^4^King Abdullah International Medical Research Center (KAIMRC), Riyadh, Saudi Arabia.

^5^Biology Department, Faculty of Science, Princess Nourah bint Abdulrahman University, Riyadh, Saudi Arabia.

^6^Biology Department, Faculty of Science, Aljouf University, Aljouf, Saudi Arabia.

^7^Zoology Department, College of Science, King Saud University, Riyadh, Saudi Arabia.

^8^School of Earth and Environmental Sciences & Research Institute of Oceanography, Seoul National University, Seoul, Republic of Korea.

^9^School of Healthcare Science, Manchester Metropolitan University, Manchester, United Kingdom.

^10^Zoology Department, Faculty of Science, Beni-Suef University, Beni-Suef, Egypt.

**Corresponding author:**

**Ayman M. Mahmoud, PhD**

Physiology Division, Zoology Department, Faculty of Science, Beni-Suef University, Salah Salim St., 62514, Beni-Suef, Egypt.

Tel.: +201144168280. E-mail: [ayman.mahmoud@science.bsu.edu.eg](mailto:ayman.mahmoud@science.bsu.edu.eg)


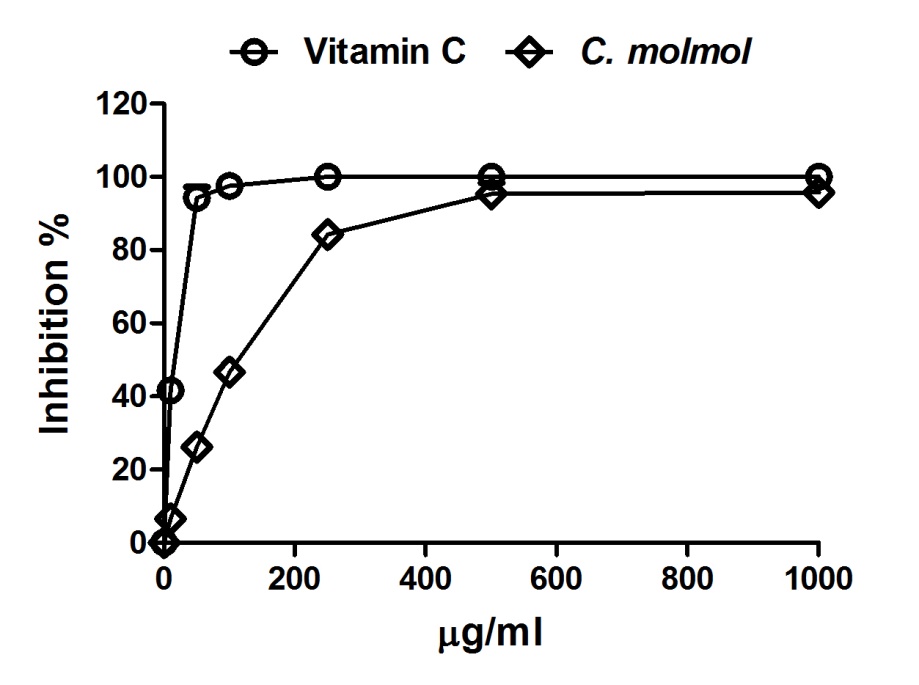


**Supplementary Figure I**: DPPH radical scavenging activity of *C. molmol* resin extract. Data are the mean values of triplicate and expressed as mean ± SEM.
